# miRNAs involved in transcriptome remodeling during pollen development and heat stress response in *Solanum lycopersicum*

**DOI:** 10.1038/s41598-020-67833-6

**Published:** 2020-07-01

**Authors:** Mario Keller, Enrico Schleiff, Stefan Simm

**Affiliations:** 1 Department of Biosciences, Molecular Cell Biology of Plants, Goethe University, 60438 Frankfurt am Main, Germany; 2 Frankfurt Institute of Advanced Studies, 60438 Frankfurt am Main, Germany; 3 Present Address: Buchmann Institute for Molecular Life Sciences, Goethe University, 60438 Frankfurt am Main, Germany; 4 Present Address: Institute of Bioinformatics, University Medicine Greifswald, 17475 Greifswald, Germany

**Keywords:** Molecular biology, Plant sciences

## Abstract

Cellular transitions during development and stress response depend on coordinated transcriptomic and proteomic alterations. Pollen is particular because its development is a complex process that includes meiotic and mitotic divisions which causes a high heat sensitivity of these cells. Development and stress response are accompanied by a reprogramming of the transcriptome, e.g. by post-transcriptional regulation via miRNAs. We identified known and potentially novel miRNAs in the transcriptome of developing and heat-stressed pollen of *Solanum lycopersicum* (tomato). The prediction of target mRNAs yielded an equal number of predicted target-sites in CDS and 3′UTR regions of target mRNAs. The result enabled the postulation of a possible link between miRNAs and a fine-tuning of transcription factor abundance during pollen development. miRNAs seem to play a role in the pollen heat stress response as well. We identified several heat stress transcription factors and heat shock proteins as putative targets of miRNAs in response to heat stress, thereby placing these miRNAs as important elements of thermotolerance. Moreover, for members of the AP2, SBP and ARF family members we could predict a miRNA-mediated regulation during development via the miR172, mir156 and mir160-family strengthening the current concept of a cross-connection between development and stress response in plants.

## Introduction

Regulatory mechanisms in the context of development or abiotic and biotic stresses are of general importance for cell proliferation and survival. In the course of evolution multiple molecular mechanisms arose to ensure organismic development and proper responses to environmental alterations^1–4^. Sessile plants are particular in their response, especially to abiotic stresses e.g. enforced by temperature fluctuations^5–8^. In times of increased weather fluctuations, it becomes important to understand the response mechanism to environmental changes and to describe the central regulatory principles in general and in heat sensitive tissues like the pollen in particular^9, 10^. The development of the latter is accompanied by major changes of the transcriptome and proteome^11–14^. Moreover, due to these changes a transition from high (early stage) to low heat sensitive (mature stage) pollen has been described^15^.

The transcriptional and translational reprogramming as onset of cellular adaptations includes post-transcriptional and post-translational events^16^. It was shown that non-coding (nc) RNAs play a major role in the cellular homeostasis^17,18^. They allow a more rapid response than achieved by reprogramming transcription, a more fine-tuned reaction with respect to the intensity of the stress response and a direct coupling between developmental and environmental situations. One group of ncRNAs with well described post-transcriptional impact are miRNAs^18^. These ~ 20 to 24 nucleotides long, single-stranded RNAs exist in animals and plants, although without any conserved miRNA family between the two kingdoms^19^. The biogenesis of miRNAs requires two cleavage steps in which a mature miRNA is released from a hairpin structure contained in the precursor RNA^18–20^. While in animals the two cleavage steps are carried out spatially separated in the nucleus and cytoplasm via Drosha and Dicer, in plants both cleavage steps are carried out in the nucleus by a single member of the Dicer-like protein family^18,20^. The actual role of miRNAs is the guidance of the RNA-induced silencing complex (RISC) to a target mRNA via complementary base-pairing, which either leads to a cleavage of the mRNA or to inhibition of translation^18,20^. It was shown that animal miRNAs preferentially guide the RISC to regions located in the 3′ UTR^21,22^, while it has been discussed that miRNAs in *Arabidopsis thaliana* guide the RISC to regions located in the coding sequence (CDS)^23^. Despite differences in their biogenesis and binding preferences, animal and plant miRNAs preferentially target mRNAs encoding transcription factors and thus regulate transcriptional networks^24,25^.

*Solanum lycopersicum* (tomato) is a model for fleshy fruit development, general developmental reprogramming and for abiotic stress reaction due to its agricultural importance world-wide^26–30^. Over the last decades a solid knowledge on chaperones, heat stress (HS) transcription factors and their complex regulatory mechanism has been established^28,31^. Interestingly, some wild accessions of tomato have a higher heat tolerance than the cultivated *Solanum lycopersicum* var. lycopersium. This change of heat sensitivity might be attributed to genomic regions lost during evolution or breeding^32,33^. In addition, alterations of alternative splicing at different temperatures have been described that might influence the heat stress response (HSR) in tomato as exemplified for pollen^34^.

The role of miRNAs in the development and stress response of tomato pollen is not well understood. A recent study provided first insights in the repertoire of miRNAs in developing pollen under non-stress and HS conditions^35^. A total of 558 miRNAs were identified, of which only 4 showed a HS-dependent abundance change. Target prediction for miRNAs yielded an enrichment of mRNAs coding for proteins classified as “protein binding”, “DNA binding”, and “Serine/Threonine kinase”. However, the putative role of the miRNAs in reprograming the transcriptome during development or in response to heat was not fully evaluated.

The absence of a broad knowledge of miRNA-mRNA interactions in developing and heat-stressed pollen motivated us to reassess the existing small RNA-seq and Massive Analysis of cDNA Ends (MACE) datasets^14,35^. Based on the bioinformatics identification of known and potentially novel miRNAs and the prediction of potential target mRNAs, we were able to predict networks of miRNA-mRNA interactions with an expected impact on development and HS response of pollen. These predicted networks provide the basis for formulating new hypotheses and planning of future molecular and biochemical analyses.

## Results

### Identification of putative miRNAs during pollen development under normal and heat stress conditions

**Figure 1:**
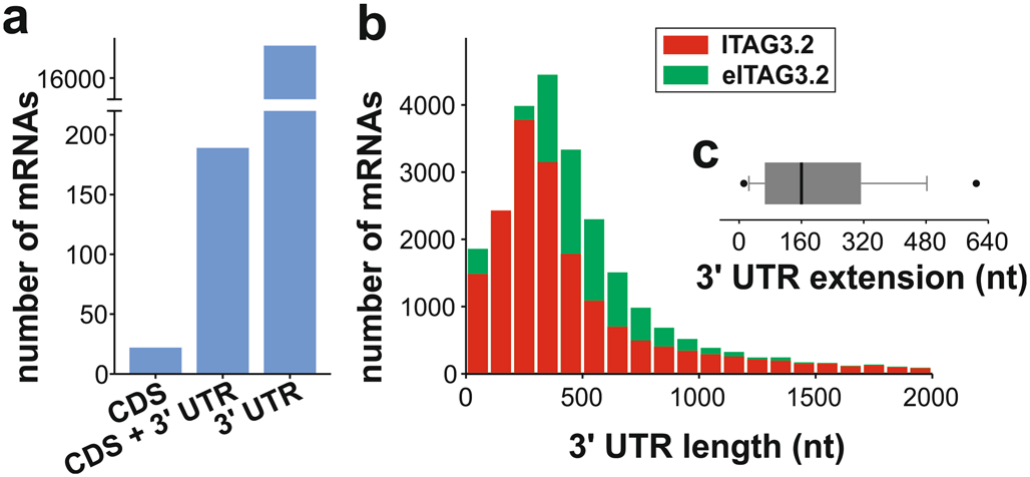
Extension of mRNA 3′ ends. (**a**) Number of mRNAs, whose CDS, CDS+ 3′ UTR or 3′ UTR were extended according to the procedure described. (**b**) 3′ UTR length distributions of ITAG3.2 (red) and eITAG3.2 annotation (green). (**c**) Distribution of 3′ UTR lengths increase between ITAG3.2 and eITAG3.2 annotations.

We aimed to establish an experimentally testable set of miRNAs involved in the molecular mechanisms contributing to the massive transcriptional and translational reprogramming during pollen development. It is discussed that many plant miRNAs target the CDS, but miRNAs targeting the 3′ UTR of mRNAs have been discovered as well^23,36^. Thus, a proper annotation of mRNA 3′ ends is mandatory for quantification of mRNA abundance and for the identification of the miRNA target sites^37^. For this purpose, the 3′ ends of all annotated mRNAs were reanalyzed based on the 18 MACE^38^ datasets, which contain sequenced mRNA 3′ ends of non- and heat-stressed pollen at different developmental stages (Supplementary Fig. 1)^14^. In total, for 17,019 of the 35,768 annotated mRNAs an extension in comparison to the current mRNA annotation was observed. These extensions affected the CDS of 22 mRNAs, the CDS and 3′ UTR of 189 mRNAs and the 3′ UTR of 16,808 mRNAs (Fig. 1a). The imbalance of the values for the different regions is a result of the exclusive 3′ end sequencing by MACE. The actual number of mRNAs with an annotated 3′ UTR changed from 18,410 (51% of all annotated mRNAs; ITAG3.2) to 25,121 (70% of all mRNAs; annotated as *extended ITAG3.2; eITAG3.2*) (Fig. 1b). The identified extensions of 3′ UTRs were in a range of 1 nt up to 2,622 nt with a median of 160 nt (Fig. 1c; Supplementary Fig. 2). Based on the annotations in eITAG3.2 it was possible to assign at least 59% of the reads of each library to mRNAs (Table 1), which served as basis for the quantification of the mRNAs.

**Table 1:** Alignment statistics of the MACE libraries.

	Sample	Replicate	Library size	Aligned reads	Reads aligned on mRNAs
Tetrads	CO	1	4,220,434	2,971,513 (70.4%)	2,516,064 (59.6%)
		2	4,548,389	3,346,633 (73.6%)	2,830,633 (62.2%)
		3	5,398,343	4,304,309 (79.7%)	3,653,033 (67.7%)
	HS	1	6,917,504	5,068,102 (73.3%)	4,136,556 (59.8%)
		2	5,690,789	4,548,590 (79.9%)	3,857,340 (67.8%)
		3	4,775,683	3,674,516 (76.9%)	3,143,600 (65.8%)
Post-meiotic	CO	1	7,221,666	6,051,418 (83.8%)	5,345,146 (74.0%)
		2	8,131,060	6,682,864 (82.2%)	5,858,333 (72.0%)
		3	5,115,229	4,311,698 (84.3%)	3,819,011 (74.7%)
	HS	1	7,283,901	6,185,931 (84.9%)	5,428,617 (74.5%)
		2	6,345,970	5,401,924 (85.1%)	4,780,752 (75.3%)
		3	2,311,857	1,935,023 (83.7%)	1,638,078 (70.9%)
Mature	CO	1	7,423,230	6,147,944 (82.8%)	5,414,995 (72.9%)
		2	5,387,727	4,625,139 (85.8%)	4,087,814 (75.9%)
		3	6,847,578	5,779,514 (84.4%)	5,062,600 (73.9%)
	HS	1	3,493,375	2,789,352 (79.8%)	2,449,650 (70.1%)
		2	4,738,745	3,833,860 (80.9%)	3,347,578 (70.6%)
		3	3,205,951	2,706,620 (84.4%)	2,355,087 (73.5%)

Shown are statistics for the MACE libraries of non- (CO) and heat-stressed (HS) tetrads, post-meiotic and mature pollen. For each biological replicate the library size, the number and percentage of aligned reads and the number and percentage of reads aligned on mRNAs is given.

**Figure 2:**
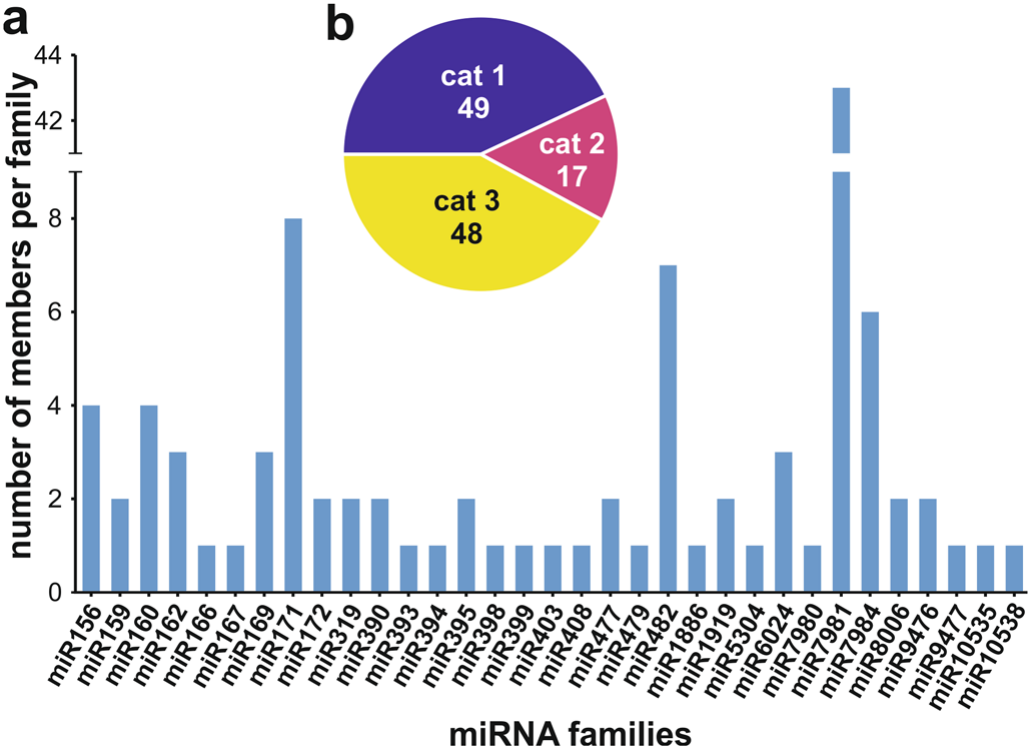
Assignment of predicted miRNAs into miRNA families. (**a**) Based on sequence similarity to hairpin sequences deposited in the miRBase, the predicted miRNAs were assigned into different miRNA families. (**b**) The assignment of a miRNA into a miRNA family was classified based on three categories (cat 1–3) as described in the result section. The number of miRNAS of each category is indicated.

**Figure 3:**
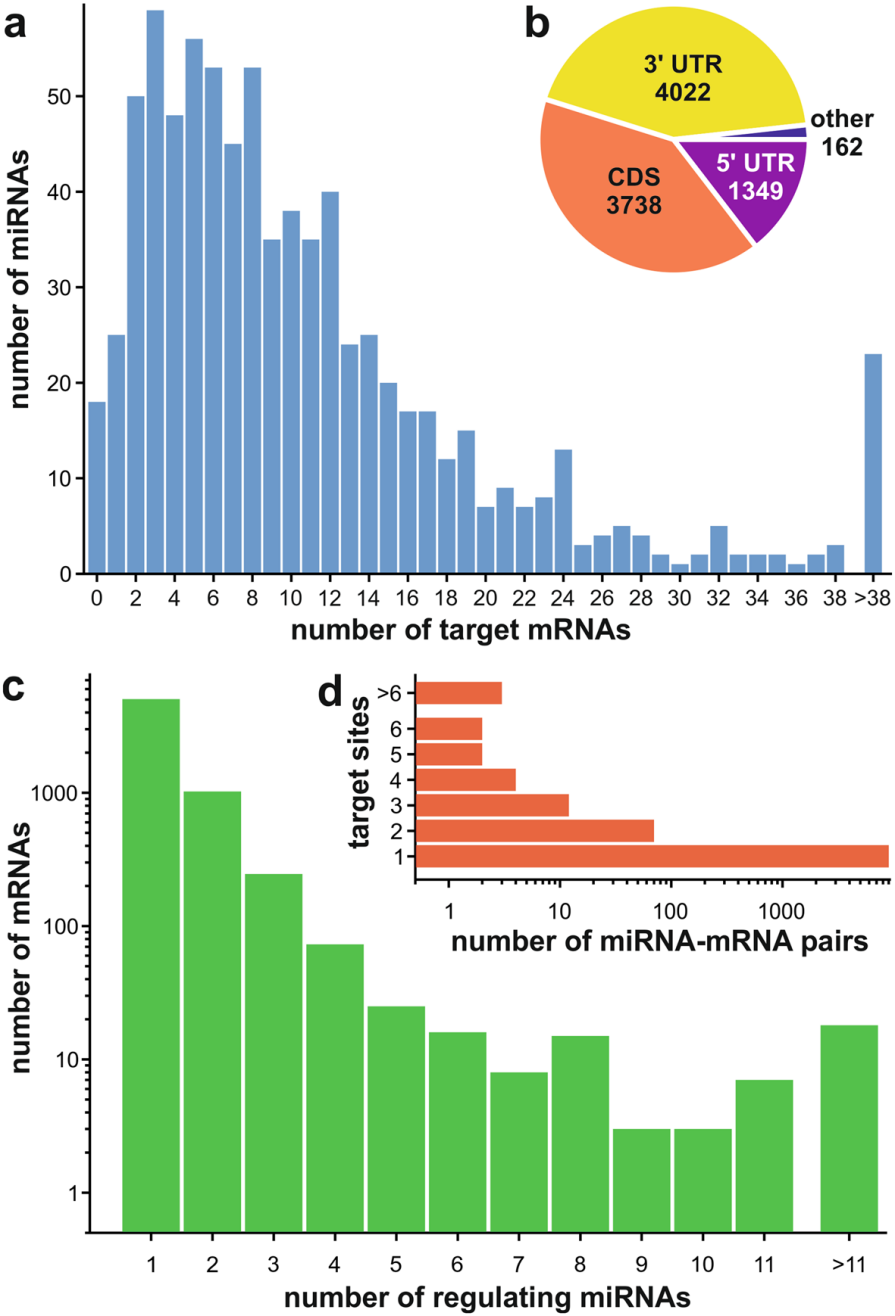
miRNA-mRNA interaction in tomato pollen stages. (**a**) Number of miRNAs with indicated number of predicted target mRNAs. (**b**) Pie chart representing the number of target sites in specific regions of the target mRNAs. (**c**) Number of mRNAs that are targeted by the indicated number of miRNAs. (**d**) Number of miRNA-mRNA pairs where the miRNA has a certain number of target sites on a single mRNA.

**Figure 4:**
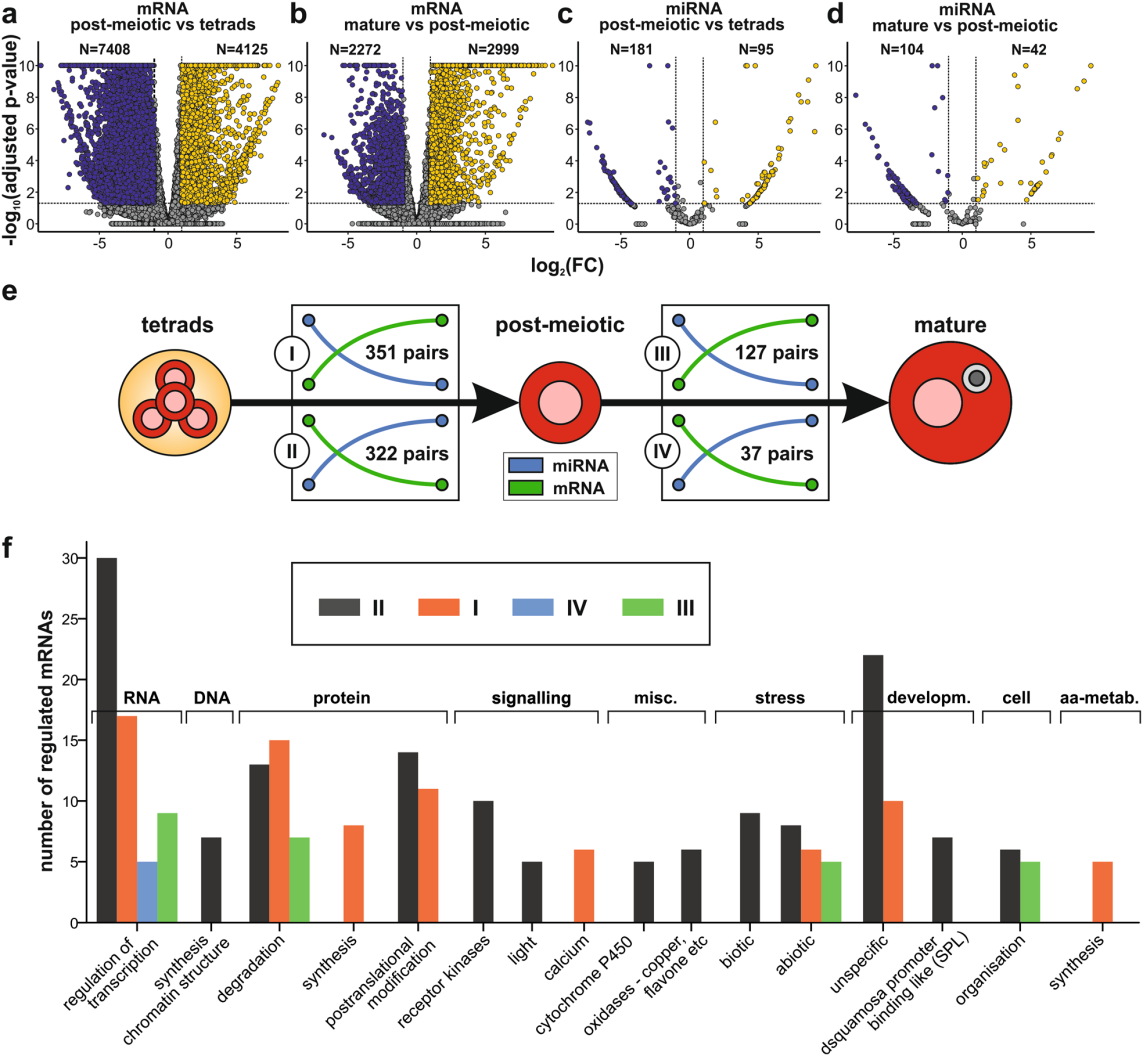
Inversely regulated miRNA-mRNA pairs during developmental transitions. (**a**–**d**) Volcano plots represent the results of differential expression analyses that were performed for mRNAs and miRNAs between successive developmental stages. For each comparison the number (N) of down- (dark blue), upregulated (yellow) and not regulated (grey) mRNAs and miRNAs is indicated. (**e**) Based on the differential expression results pairs of miRNAs (light blue) and mRNAs (green) identified that are inversely regulated during the transition from tetrads to post-meiotic pollen as well as during the transition from post-meiotic to mature pollen. (**f**) Assignment of inversely regulated mRNAs to MapMan terms of the second hierarchy level. Here, only Map Man terms with at least 5 assigned mRNAs are shown.

A total of 790 potential miRNAs were identified by the bioinformatics pipeline established in the small RNA-seq libraries (Supplementary Fig. 3a; Supplementary Table 1). They were named in ascending order based on their lexicographically ordered nucleotide sequences (solyc-miR001 to solyc-miR790). The identified potential miRNAs were assigned to miRNA families by a search against the hairpin sequences deposited in the miRBase^39^. Based on their best match they were assigned into four categories (Fig. 2; Supplementary Fig. 3b): (1) the 5′ end overlaps with the seed region of an annotated miRNA on the hairpin (49 of the identified potential miRNAs; Fig. 2b), (2) partial overlap with an annotated miRNA on the hairpin (17 miRNAs; Fig. 2b), (3) overlap with hairpin but with no miRNA (48 miRNAs; Fig. 2b) and (4) no overlap with a hairpin (676 miRNAs; Supplementary Table 2). In total, 33 different miRNA families based on the miRBase were observed with sizes ranging from one (e.g. miR166) up to 43 members (miR7981 family). The miR482 family contains with six out of its seven members the highest fraction on category 1 members. For subsequent analyses we used all putative 790 miRNAs, regardless if they were assigned to a known miRNA family. This strategy ensured that we do not exclude miRNAs of pollen, which are not yet deposited in the miRBase due to the low number of pollen miRNA studies.

A miRNA target prediction was performed to identify mRNAs regulated by the 790 miRNAs. The prediction was performed with psRNATarget using the miRNAs and the cDNA sequences of eITAG3.2 as input (Fig. 3). In total, 9,271 interactions were predicted that are distributed across 9,115 miRNA-mRNA pairs (Supplementary Table 3). The higher number of interactions than miRNA-mRNA pairs arises from 93 miRNA-mRNA pairs that interact via more than one target site (Fig. 3d). The number of target mRNAs per miRNA is in a range of zero to more than 38 mRNAs per miRNA (Fig. 3a). A preference for binding in the 3′ UTR (4,022 target sites) and CDS (3,738 target sites) of the target mRNAs was observed for the identified 790 miRNAs, while predicted binding to the 5′ UTR (1,349 target sites) was less frequent (Fig. 3b). Among the 9,115 predicted miRNA-mRNA pairs there are 6,486 different mRNAs, which implies that a single mRNA may be targeted by more than one miRNA^40^. However, the vast majority of the 5,048 mRNAs is targeted by only one of the 790 predicted miRNAs (Fig. 3c).

### Putative miRNAs are predicted to regulate transcriptional-networks during pollen development

Next, we aimed to identify miRNAs that could regulate mRNAs in a developmental context. Due to the lack of experimental information on protein abundance, we focused on the identification of miRNAs among the 790 identified miRNAs that putatively induce the degradation of their target mRNAs. For that reason, we identified all miRNAs and mRNAs that are differentially abundant in two consecutive developmental stages.

The differential transcript abundance analyses for miRNAs and mRNAs were performed with DESeq2 between post-meiotic pollen and tetrads as well as between mature and post-meiotic pollen (Fig. 4a–d). The number of mRNAs and miRNAs with altered abundance, is higher between post-meiotic pollen and tetrads than between mature and post-meiotic pollen. Interestingly, more mRNAs appear to be reduced than enhanced in their abundance in post-meiotic pollen when compared to tetrads and the opposite could be observed for the comparison between mature and post-meiotic pollen. The number of predicted miRNAs with reduced abundance exceeds the number of miRNAs with enhanced abundance for each transition. The predicted miRNA-mRNA pairs were afterwards screened for those showing opposite profiles during the two analyzed developmental transitions (Fig. 4e; Supplementary Table 4). Out of all miRNA-mRNA pairs 351 with decreased miRNA and increased mRNA abundance during the transition from tetrads to the post-meiotic state were identified. Moreover, 322 predicted pairs showed the opposite behavior during the same transition. During the transition between post-meiotic and mature stage 127 predicted pairs with a higher miRNA and lower mRNA abundance, as well as 37 predicted pairs with the opposite behavior were observed.

The mRNAs of the inversed pairs were functionally classified based on the second hierarchy level of the MapMan ontology (Fig. 4f; Supplementary Table 5) to infer their putative potential in pollen development. For the transition between tetrads and post-meiotic pollen we identified 16 functional subclasses in nine main categories with at least five genes. The main categories RNA, protein, signaling and development are most prominent. For 13 terms a down- (Fig. 4f grey) and for eight terms an upregulation (Fig. 4f orange) could be observed in post-meiotic pollen compared to the tetrads stage. In the comparison between mature and post-meiotic stage only four terms (‘RNA. regulation of transcription’, ‘protein.degradation’, ‘stress.abiotic’, ‘cell.organisation’) contain at least five genes. Out of all, ‘RNA.regulation of transcription’ was the term with the highest number of assigned mRNAs for all four sets of inversely regulated pairs. A closer look on the functional description of these mRNAs revealed that many of them encode for transcription factors and 61 TF encoding mRNAs out of 21 TF families have an altered RNA abundance during at least one developmental transition (Supplementary Table 6). Among the different putative miRNAs that are predicted to regulate TFs during the two developmental transitions are eight miRNAs that perfectly or nearly perfectly match a known miRNA (category 1; Supplementary Table 1; Supplementary Fig. 3b).

### Putative miRNAs are predicted to be involved in the heat stress response of pollen

**Figure 5:**
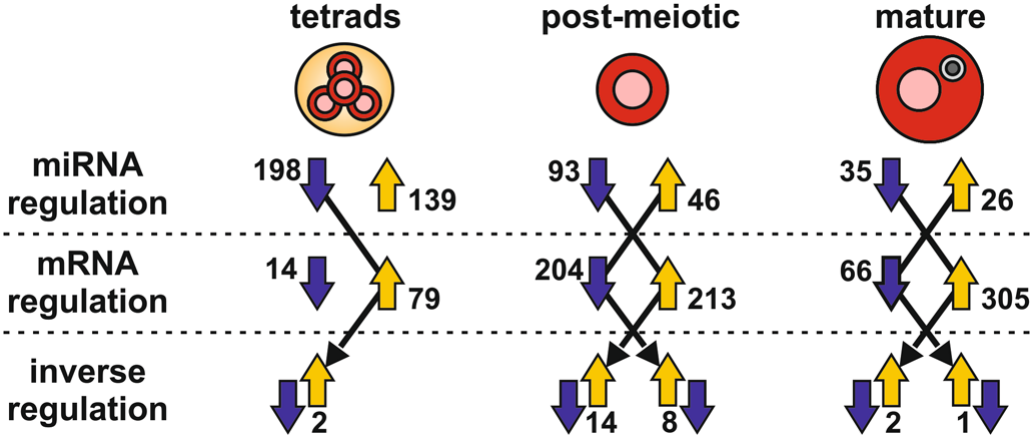
The role of miRNAs in the heat stress response of pollen developmental stages. Differentially regulated miRNAs and mRNAs in response to HS were determined by differential expression analyses, followed by the identification of inversely regulated miRNA-mRNAs for tetrads, post-meiotic and mature pollen.

Next, we aimed to assign a role of miRNAs in the HSR of the pollen at different developmental stages. For this purpose, differential expression analyses between the non- and heat-stressed samples were performed individually for each developmental stage for the predicted miRNAs and their predicted mRNA targets (Fig. 5). The results show that the number of differentially regulated mRNAs in response to HS is much smaller than the number of differentially regulated mRNAs during the developmental transitions. We identified only two predicted miRNA-mRNA pairs with an inverse regulation in tetrads, 22 with an inverse regulation in post-meiotic pollen and three with an inverse regulation in mature pollen. As two of the identified pairs were identified in post-meiotic and mature pollen, there are 25 predicted pairs with an inverse regulation in at least one developmental stage. Further, two of the miRNAs target the same mRNA, which is why the 25 pairs contain 24 different mRNAs (Supplementary Table 7). The regulon will be discussed below.

## Discussion

### The miRNA complexity of developing and heat-stressed tomato pollen

Reannotation of mRNA 3′ ends led to the extension of existing 3′ UTRs for 6,711 mRNAs (Fig. 1). The average 3′ UTR length across all annotated mRNAs is 417nt (eITAG3.2), which is between the average 3′ UTR length of *Arabidopsis thaliana* (242nt) and rice (469nt)^41^. Using this revised annotation (eITAG3.2), we predicted that the 790 putative miRNAs identified by small RNA sequencing regulate a set of 6,486 different mRNAs throughout pollen development and HSR. Please note, it cannot be stated if our results are pollen specific or represent general mechanisms for plant tissues because there are only few studies focusing on plant wide tissue specific miRNA regulation^42–44^. However, the number of 790 identified putative miRNAs in tomato pollen is larger than the 486 observed miRNAs in developing pollen of diploid and autotetraploid rice^45^. The search of the 790 putative miRNAs against the hairpin sequences deposited in the miRBase allowed the assignment of 114 miRNAs to 33 different miRNA families (Fig. 2). Out of the 114 miRNAs, 49 miRNAs matched a mature miRNA on the hairpin sequence (Supplementary Fig. 3b category 1). The family with the highest category 1 assignment is miR482 (Supplementary Table 2). This miRNA family has been described in 2-week-old seedlings of tomato, where six members were identified (slymir482a, b, f)^46^. Three of the latter were identified in pollen as well (solyc-miR642, solyc-miR643 and solyc-miR646). Thus, we propose 790 putative miRNAs to be involved in pollen development or stress response in tomato.

### The relation of the predicted miRNAs and the transcriptional networks during pollen development

We predicted that on average 11 to 12 mRNAs are targeted by a single miRNA. This number is higher than the number predicted for rice (~ 7 targets per miRNA) or maize (~ 8 targets per miRNA), but comparable to the number proposed for Chinese cabbage (10 to 11 targets per miRNA)^47–49^. Despite the initial hypothesis that plant miRNAs preferentially target the CDS of target mRNAs^38^, our prediction resulted in a slightly higher fraction of target sites in the 3′ UTR than in the CDS, which was also described for animal miRNAs^21^ (Fig. 3). The high fraction of 3′ UTR binding sites might either be a result of the 3′ UTR extension, which may have led to an inclusion of additional miRNA binding sites previously missed, or might reflect a distinct regulatory mode of pollen-specific miRNAs when compared to miRNAs specific for other tissues. However, this notion has to be challenged by additional large scale miRNA analysis of different tissues and plants and experimental validation of the individual binding pairs by alternative methods.

**Figure 6:**
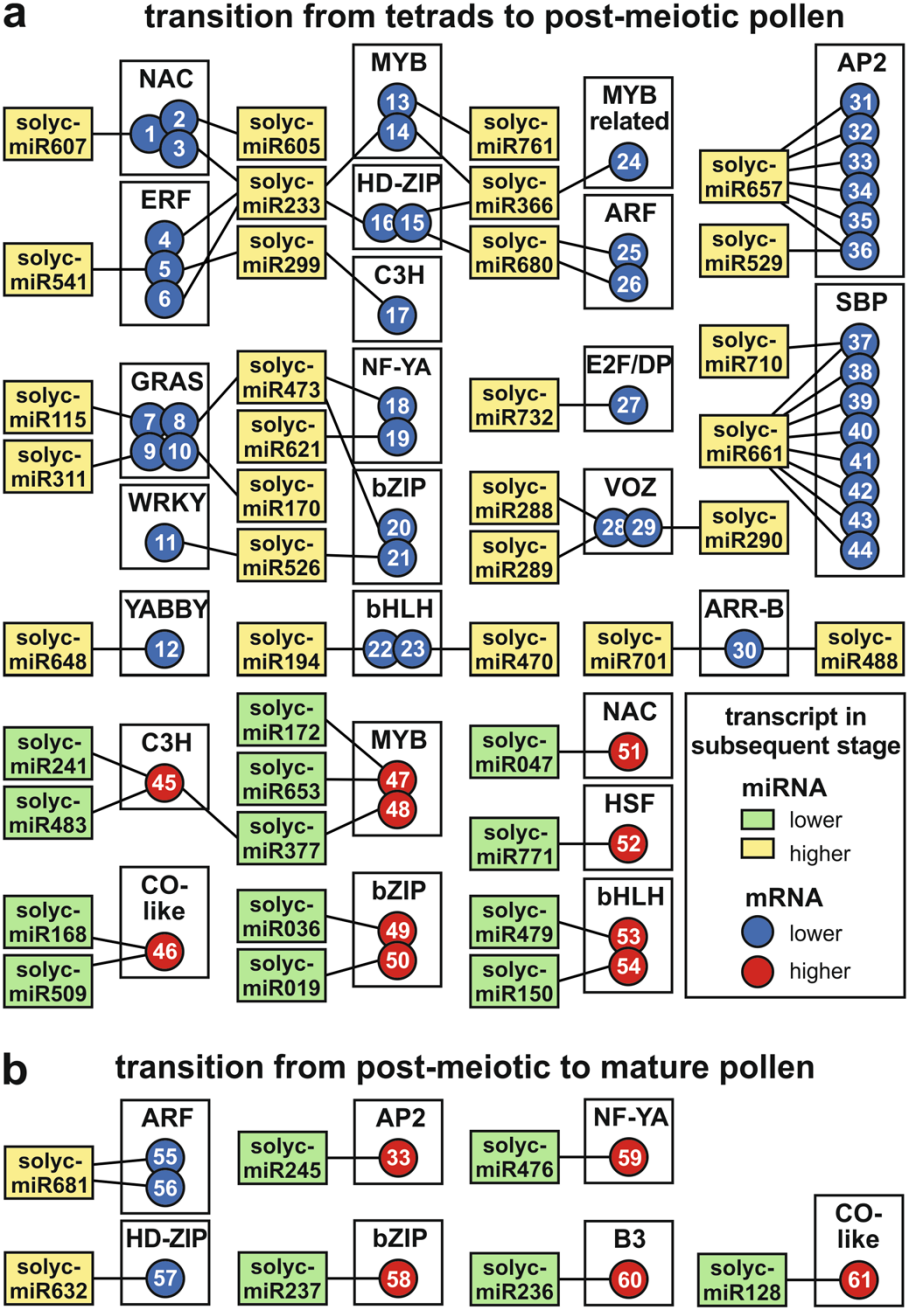
miRNA-mediated regulation of transcription factors during pollen development. (**a**, **b**) Shown are interactions between miRNAs (rectangles) and mRNAs (circles) where the miRNA and mRNA are inversely regulated during the transition from tetrads to post-meiotic pollen (**a**) and during the transition from post-meiotic to mature pollen (**b**). The regulation during the transition is indicated via the color of the rectangles and circles and is either a downregulation (green and blue) or an upregulation (yellow and red). Abbreviations are given in discussion or are: GRAS: gibberellic acid sensitive (GAI), repressor of GAI (RGA) and scarecrow (SRC); HD-ZIP: Homeodomain-leucine zipper.

Many of the mRNAs of inversely regulated predicted miRNA-mRNA pairs would be involved in the regulation of RNA synthesis (Fig. 4f). This is a known phenomenon in plants^25^ and was shown to be important for different developmental processes, such as root, seed and also pollen development^50–52^. In total, 61 TF encoding mRNAs are predicted to be regulated via miRNAs during the two analyzed developmental transitions (Fig. 6; Supplementary Table 6). Remarkably, 43% of the TFs are predicted to be regulated by 3′ UTR targeting miRNAs and 48% by CDS targeting miRNAs (Supplementary Table 6). Out of the putative regulating miRNAs, eight show a perfect or nearly perfect match to an annotated miRNA of a hairpin sequence (category 1). Two of these miRNAs are solyc-miR680 and solyc-miR681 (miR160 family), which target mRNAs encoding for members of the Auxin responsive factor (ARF) TF-family during the first and second transition, respectively. In both cases, the upregulation of the miRNA as judged from transcript abundance changes correlates with a downregulation of the ARF mRNAs. Our prediction is consistent with previous experimental reports as regulation of ARF mRNAs by miR160 family members was described for other plant species, such as *A*. *thaliana* and rice^53,54^. There, miR160-resistant versions of ARFs led to dramatic growth and developmental defects, which indicates that the fine-tuned regulation of ARFs via miR160 might be essential for proper pollen maturation. This hypothesis is supported by the observation that *A. thaliana arf17* mutant plants showed pollen wall patterning defects and pollen degradation^55^. Interestingly, the ARF17 mRNA is downregulated during the second transition (Fig. 6 number 56), which suggests also an important role of this transcription factor in tomato pollen.

**Table 2:** Validation of cleavage events on TF cDNAs in published degradome sequencing data.

TFF	mRNA ID	TS on cDNA	CS on cDNA	Sequencing tags (cleavage site / whole cDNA)				Nucleotide composition
				5 DPA	MG	Br	RR	
AP2	Solyc02g093150.3.1	1,560–1,580	1572	170/83	133/379	109/211	23/71	_GCTG_CTGCAGCA**T**CATCAGGATTCC_CTCA_
	Solyc03g044300.3.1	1,366–1,386	1,384	127/267	164/364	3,353/5,359	465/1582	_ACTG_CTGCAGCA**T**CATCAGGATTCC_CCCA_
	Solyc02g064960.3.1	1764–1784	1744	170/304	133/165	109/300	23/77	_ACTG_CTGCAGCA**T**CATCAGGATTCC_CTCA_
	Solyc06g075510.3.1	1,348–1,368	1522	146/233	23/86	3/5	1/5	_TGCA_CGGCAGCA**T**CATCAGGATTCT_TCAT_
	Solyc09g007260.3.1	1624–1644	1523	81/337	8/40	–/–	1/4	_TCTA_CTGCAGCA**T**CATCAGGATTCG_CTAA_
	Solyc10g084340.2.1	2,182–2,202	1,252	164/210	13/22	1/8	0/3	_TCTA_CTGCAGCA**T**CATCAGGATTCG_GAAA_
SBP	Solyc07g062980.3.1	443–462	448	–/–	1/6	–/–	–/–	_TGAA_ATGCTTACTC**T**CTTCTGTCA_GCCC_
	Solyc05g015510.3.1	2,555–2,574	2,962	3/520	50/895	55/633	38/652	_CCTC_GTGCTCTCTCT**C**TTCTGTCA_ACTA_
	Solyc05g015840.3.1	900–919	760	1/39	1/18	–/–	–/–	_GATC_GTGCTCTCTCT**C**TTCTGTCA_TCAG_
	Solyc10g078700.2.1	3,614–3,633	775	24/660	51/117	1/7	1/4	_AGTG_GTGCTCTCTCT**C**TTCTGTCA_AATC_
	Solyc12g038520.2.1	1,431–1,450	981	3/69	12/41	1/10	4/8	_GGCT_GTGCTC**T**CTCTCTTCTGTCA_TCTC_
ARF	Solyc11g069500.2.1	1836–1857	1,313	17/433	42/634	145/524	10/307	_ACTG_CAGGCATACAGG**G**AGCCAGGCA_TGCT_

Degradome sequencing data^64^ for TF (TF, column 1) mRNAs (column 2) predicted to be regulated by miRNAs (column 3: target site (TS); eITAG3.2) were analyzed. For each mRNA the cleavage site (column 4—CS; ITAG2.3) and sequencing tag information is indicated^64^. Sequencing tag information includes the number of tags at the crosslink position and on the whole cDNA for four stages of tomato fruit development (5 days post-anthesis (5 DPA), mature green (MG), breaker (Br) and red ripe (RR)). In the last column the nucleotide composition in the target site region is shown, whereby large letters indicate the target site region and bold letters the identified cleavage site^64^. Differences between target and cleavage site positions are due to differences in the annotation releases (eITAG3.2 and ITAG2.3).

Moreover, solyc-miR529 and solyc-miR657 (miR172 family) are enhanced during the first transition, which coincides with a reduction of their predicted target mRNAs coding for members of the Aptella 2 (AP2) TF-family (Fig. **6**). Both miRNAs have one AP2 target in common, while solyc-miR657 targets five additional AP2s. Consistent with our observation, a miR172 family member is required for the regulation of two AP2 encoding mRNAs in mature pollen of *Brassica campestris*^56^. Complementary, our results indicate that the miR172-mediated regulation of AP2 members starts in post-meiotic pollen.

In addition, members of the Squamosa promoter binding protein (SBP) TF-family are likely downregulated in a miRNA-dependent manner during the first transition (Fig. 6). Eight SBP mRNAs are likely regulated by solyc-miR661 (miR156 family). Such miR156-mediated regulation of SBPs appears to be globally conserved in plants, as it has been described e.g. for *A*. *thaliana*, barley and pear^40,57–60^. Consistent with our discovery of solyc-miR661, overexpression of miR156 in *A*. *thaliana* leads to a downregulation of SBP encoding mRNAs with a simultaneous decrease in pollen grain production^58^.

There are also miRNAs downregulated during developmental transitions coinciding with upregulation of their predicted targeted mRNAs. One of these miRNAs is solyc-miR476 (miR169 family) targeting a member of the Nuclear factor-YA (NF-YA) TF-family. Again, this is consistent with experimental evidence as a miR169-mediated regulation of NF-YA encoding mRNAs was discovered to be involved in root development and adaptation to abiotic stresses in *A. thaliana* and tomato^61^. In tomato it was shown that miR169 is accumulated in response to drought stress, while NF-YA mRNAs are downregulated in this condition. Overexpression of miR169 in tomato led to a downregulation of NF-YA mRNAs as well as to an enhanced drought tolerance^62^. According to our bioinformatics results, the detected miR169 member represses the mRNA levels of the NF-YA mRNA in the tetrad and post-meiotic stage, while upon the transition from post-meiotic to mature pollen the miRNA is less abundant and the NF-YA mRNA enhanced. This behavior might contribute to the drought tolerance of the earlier developmental stages and fits to the concept of developmental priming, where the accumulation of stress-responsive proteins is thought to protect early developmental stages against sudden stresses^63^.

Further support of our predictions is provided by the discovery of miRNA-dependent cleavage sites of AP2, SBP and ARF family members. These cleavage sites were identified by degradome sequencing of RNA isolated from tomato fruits at different developmental stages (Table 2)^64^. After accounting for changes in the annotations (eITAG3.2 and ITAG2.3), we identified for all three TF-families cleavage sites within the predicted target sites, which supports our target site postulation. Moreover, it is worth mentioning that similar to our predictions the AP2s, SBPs and the ARF are regulated by the miR172, mir156 and mir160 family, respectively, in developing tomato fruits^64^. This observation suggests that miRNAs are regulators of developmental processes.

### miRNA-mediated regulation during development is reversed by heat stress

**Figure 7:**
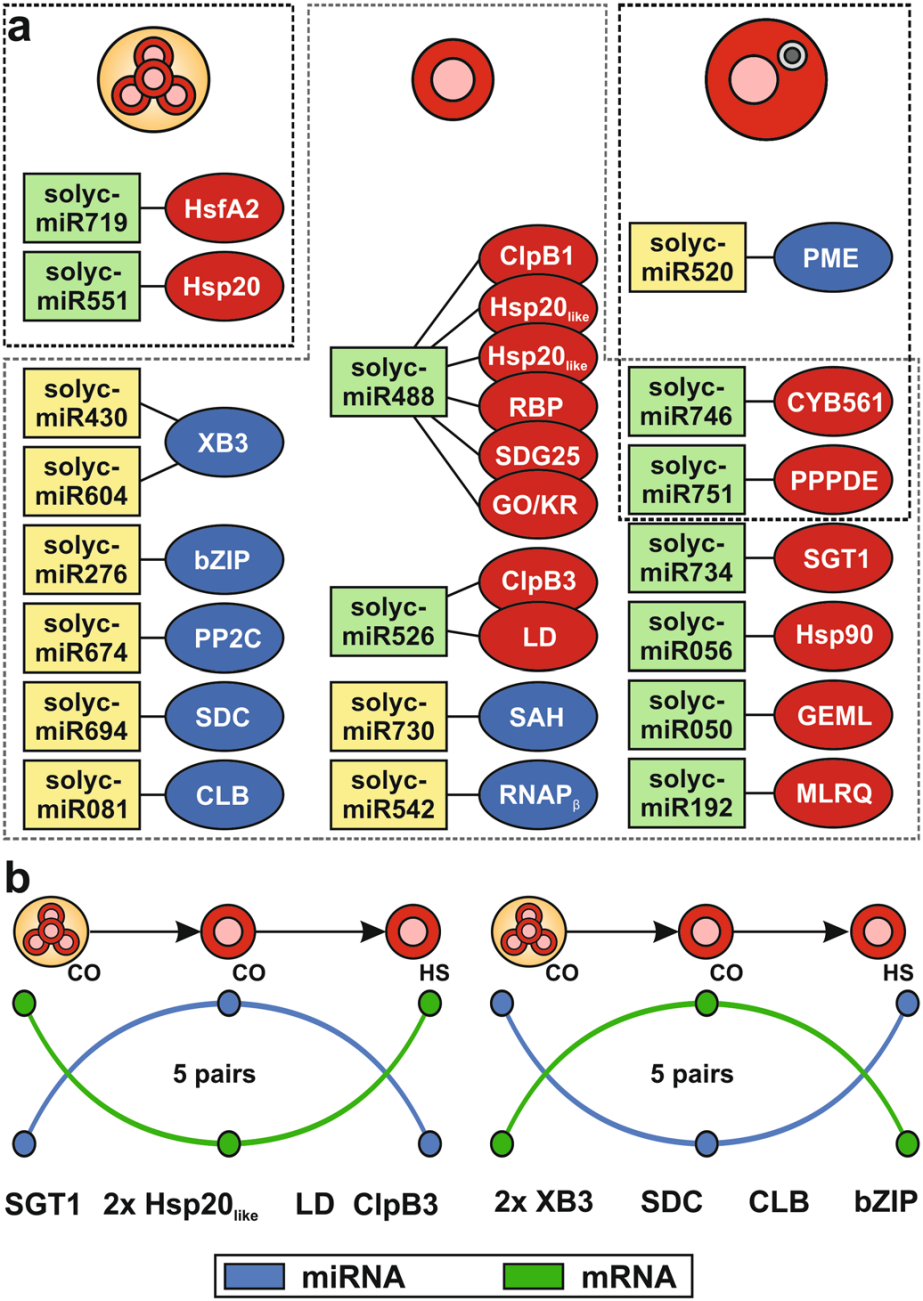
miRNA-mediated regulation during heat stress related to pollen development. (**a**) Shown are interactions of miRNAs (rectangles) and mRNAs (circles) that are inversely regulated in response to HS in the three analyzed stages (dashed lines). The regulation is indicated via the color of the rectangles and circles as in Fig. 6 and is either a downregulation (green and blue) or an upregulation (yellow and red). (**b**) Shown are simplified profiles of miRNAs (blue) and mRNAs (green) that are inversely regulated during the transition from tetrads to post-meiotic pollen and when HS is applied on the post-meiotic pollen.

The adaptation of transcriptomes in response to stresses is regulated by multiple mechanisms including mRNA degradation mediated by miRNA targeting^17,20^. We identified 25 putative miRNA-mRNA pairs with an inverse regulation in at least one developmental stage in response to HS (Fig. 7a), 12 thereof represent pairs with 3′ UTR based miRNA regulation (Supplementary Table 7). Many upregulated mRNAs of inversely regulated pairs predicted by our methodology encode for proteins that play a central role in plant HSR. For instance, mRNAs encoding HsfA2 and a member of the Hsp20 family are upregulated in heat stressed tetrads, while their predicted corresponding miRNAs are downregulated (solyc-miR719 and solyc-miR551). Interestingly, this is consistent with the observed importance of HsfA2 thermotolerance in meiotic pollen in tomato^65^. Similarly, in post-meiotic pollen mRNAs encoding ClpB1 and ClpB3 of the Hsp100 family, an Hsp90 and two putative members of the Hsp20 family are upregulated in response to HS, while the level of the putatively targeting miRNA is reduced (solyc-miR719, solyc-miR551, solyc-miR488 and solyc-miR526). Such miRNA-mediated regulation of Hsp or Hsf mRNAs was recently described in radish and French bean^66,67^. Remarkably, the predicted miRNAs targeting Hsf and Hsp mRNAs could not be assigned to a miRNA family. However, solyc-miR526 was also identified in *Solanum pennellii*, which is a wild relative of *S*. *lycopersicum*^68^. Strikingly, the miRNA in *S*. *pennellii* is predicted to target a Hsp100 family mRNA (Sopen02g033340.1) as well.

Next to the known regulators of the plant HSR, two mRNAs are upregulated in post-meiotic as well as mature pollen, while in both developmental stages the corresponding miRNAs (solyc-miR746 and solyc-miR751) are downregulated upon HS. The mRNAs encode for a Cytochrome b561-related family protein (CYB561) and a PPPDE putative thiol peptidase family protein (PPPDE). So far, the exact role of CYB561s in the plant stress response remains to be established. However, it was suggested that CYB561s support various physiological functions, including stress defense^69^. In contrast, a role of PPPDEs in the stress response of plants has, to our knowledge, not been demonstrated yet.

Worth mentioning, 10 of the 22 putative miRNA-mRNA pairs that show an inverse regulation in post-meiotic pollen upon HS are also inversely regulated during the transition from tetrads to post-meiotic pollen, but with reversed behavior (Fig. 7b). Two mRNAs downregulated during the developmental transition and upregulated in response to HS encode a member of the Hsp20 family and ClpB3 of the Hsp100 family, respectively. Thus, in tetrads as well as in response to HS the accumulation of mRNAs of these Hsps is required, while in post-meiotic pollen the accumulation is not necessary. The accumulation of mRNAs encoding stress responsive proteins in tetrads might reflect a developmental priming, which is thought to protect them against sudden stresses^13,63^.

One of the mRNAs upregulated during the developmental transition but downregulated in response to HS encodes for a protein annotated as ankyrin repeat domain protein. Ankyrin repeat domain containing proteins are involved in pollen development as shown for lily^70^ where the protein is required for proper pollen germination and pollen tube growth. Another mRNA upregulated during the developmental transition but downregulated in response to HS encodes a bZIP TF. Overexpression of a dominant-negative form of a bZIP TF in tobacco leads to impaired pollen development^71^. Thus, we postulate that the miRNA-mediated downregulation of the ankyrin repeat protein or the bZIP TF upon HS would also have negative effects on the development of the tomato pollen.

In summary, we propose that miRNA-mRNA interactions occur during pollen development in a stage transition specific manner, which for the individual cases needs to be confirmed by in depth biochemical and molecular analyses. Some of the predicted interactions are directly linked to the developmental process, while others would contribute to the developmental priming, which is thought to protect the pollen against sudden stresses and affects majorly TFs. In addition, we provide bioinformatics evidence that HSR in pollen is regulated by miRNA-based mechanisms. However, if this holds true, it occurs to a lesser extent than during developmental transitions and miRNA based HS responses would have the greatest influence in the post-meiotic pollen-stage. Moreover, many of the postulated miRNA based HS responses are inversed regulations of those found during development, which suggests that developmental delay is part of the HSR mechanism in pollen. Further, these inverse regulations are affecting major components of the HSR like ClpB3, small Hsps and bZIP and by this could contribute to a major extent.

## Methods

### High-throughput datasets

The small RNA-seq and MACE datasets used are published^35^ and are available in the Array Express repository (accession E-MTAB-3830) and in the SRA (submission: SUB6166902) and BioProject (accession PRJNA559888), respectively. Both datasets originate from the same RNA samples that were isolated from non- or heat-stressed *S. lycopersicum* (cultivar Red Setter) pollen at tetrad, post-meiotic or mature pollen stage. All experiments were performed in biological triplicates.

### Read alignment of the MACE libraries

The 18 MACE libraries were aligned to the reference genome of *S. lycopersicum* (SL3.0; cultivar Heinz) with STAR (version 2.6.1a)^72^ using the following adjustments of default parameters: –alignSJoverhangMin 10, –alignSJDBoverhangMin 5, –outFilterMultimapNmax 1, –outFilterMismatchNmax 2, –alignIntronMin 35, –alignIntronMax 6,000 and –outSAMtype SAM. Limiting the maximum number of mismatches to 2 was based on the fact that that nucleotide divergence between cultivar Heinz and the wild relative *S. pimpinellifolium* is only 0.6%^73^ and we expect the difference between the cultivars Red Setter and Heinz to be even smaller.

### Extension of mRNA 3′ ends

For the extension of mRNA 3′ ends a self-developed workflow was established (Supplementary Fig. 1). In a first step the alignments of the 18 MACE libraries were pooled to obtain a higher coverage. Afterwards the continuous and spliced alignments were used to make a de novo reconstruction of mRNA 3′ ends. Here, continuously covered nucleotides were first merged to exons, followed by the introduction of splice junctions. In the case of overlapping splice junctions (e.g. due to alternative 5′ or 3′ splice sites) the junction with the highest coverage was considered. Further, splice junctions, whose coverage was lower than the average coverage of the nucleotides that would be skipped by the junction, were removed. Afterwards, the reconstructed 3′ ends were superimposed on annotated mRNAs of *S. lycopersicum* (version ITAG3.2). If possible, the annotated mRNAs were extended based on the superimposed 3′ ends (extended ITAG.3.2). As a final step the extended mRNA regions were classified as CDS and/or 3′ UTR.

### Identification of miRNAs in the small RNA-seq libraries

The identification of miRNAs was based on a self-developed workflow (Supplementary Fig. 3a). The reads of the 18 small RNA-seq libraries were first filtered for those reads with a length between 18 and 24 nucleotides. Subsequently, identical reads were collapsed, which means that the read sequence was present only once but the abundance stored for further processing steps. Reads with an abundance of only one were removed. To remove potential contaminations with other RNA types than miRNAs, the reads were searched against all sequences deposited in the Rfam database (accessed January 22, 2018)^74^ and only reads without matches or matches to miRNAs were kept. Subsequently, the reads were aligned to the reference genome of *S. lycopersicum* using bowtie2^75^ using the following adjustments of default parameters: -D 20, -R 3, -L 10, -i S,1,0.50 and -k 20. Alignments overlapping exons of the extended ITAG3.2 were removed in the next step. Based on the assumption of a characteristic pre-miRNA hairpin structure, for each aligned read two genomic regions were excised. The first region included the alignment position as well as 70 nt upstream and 20 nt downstream, which should cover a positioning of the miRNA in the 3′ arm. Similarly, a positioning in the 5′ arm was covered by the excision of the alignment position and 20 nt upstream and 70 nt downstream. The excised regions of each aligned read were afterwards used as input for RNAfold of the Vienna RNA package^76^. Aligned reads were considered as mature miRNAs if at least one of their excised regions fulfilled the following five criteria: (i) the aligned read had to be positioned in a hairpin loop structure, (ii) the hairpin loop has to cover at least 75% of the excised region, (iii) the aligned read must not be located in the loop, (iv) the loop has a minimal length of 4 nucleotides and (v) the minimal free energy of the predicted structure is lower than − 35 (kcal/mol). To ensure reproducibility all miRNAs not detected in all three biological replicates were removed. The final miRNAs were named in ascending order based on their lexicographically ordered nucleotide sequences (solyc-miR001 to solyc-miR790).

### Family assignment of detected miRNAs

To assign the detected miRNAs to families, they were in a first step aligned against all hairpin structures deposited in the miRBase (accessed November 13, 2018)^39^ with bowtie2 using the following adjustments of default parameters: –norc -D 20 -R 3 -N 1 -L 5 -i S,1,0.50 -p 3 -k 300. For further steps, for each miRNA only the best alignments were kept. The most abundant family among the best matching hairpins was used as the family for the miRNA. Further, the family assignments were classified based on four categories: (1) the miRNA 5′ end overlaps the seed region of an annotated miRNA on the hairpin, (2) the miRNA has only a partial overlap with an annotated miRNA on the hairpin, (3) the miRNA overlaps with the hairpin but with no annotated miRNA on the hairpin and (4) the miRNA has no overlap with an hairpin (no family assignment).

### Identification of targeted mRNAs and their functional classification

The prediction of miRNA targets was done with psRNATarget^77^ by using the detected miRNAs and the mRNA sequences of the extended ITAG3.2 as input and allowing up to 500 targets per miRNA. Predicted miRNA-mRNA pairs with an expectation value greater than three and translational inhibition as inhibition mode were removed. mRNAs targeted by at least one miRNA were afterwards functionally characterized based on the MapMan ontology by submitting the corresponding protein sequences (ITAG3.2) to the Mercator web server^78^. All functional analyses in this study are based on the second hierarchy level (e.g. protein.synthesis), which was available for most of the mRNAs and sufficient for a functional assignment. Further, mRNAs were assigned to transcription factor families based on the *S. lycopersicum* transcription factor list in the PlantTFDB (accessed December 3, 2018)^79^.

### Differential expression analysis of miRNAs and mRNAs

To identify miRNAs and mRNAs that are differentially regulated during a developmental transition (post-meiotic vs tetrads and mature vs post-meiotic) or in response to HS, the read counts of the samples were used as input for DESeq2 (version 1.16.1)^80^. The read counts of the miRNAs were obtained in the collapsing step, where identical reads were stored as a single read, while the read counts of the mRNAs were generated with htseq-count (version 0.6.0)^81^ by using the STAR alignments and the extended ITAG3.2 annotation as input.

### Validation of miRNA target sites by published degradome sequencing data

For identification of the presence of cleavage sites in selected target mRNAs we compared the nucleotide sequences comprising the target and experimental discovered cleavage sites^64^. The nucleotide sequence of the target site and its surrounding region (4 nts up- and downstream) was compared to the nucleotide sequence of the cleavage site and its surrounding region. If there was 100% similarity between the two sequences (length between 28 and 30 nts), we considered the target site as verified.

## Supplementary information

Supplementary file1 (PDF 778 kb)

Supplementary file2 (XLSX 221 kb)

Supplementary file3 (XLSX 10 kb)

Supplementary file4 (XLSX 585 kb)

Supplementary file5 (XLSX 60 kb)

Supplementary file6 (XLSX 19 kb)

Supplementary file7 (XLSX 15 kb)

Supplementary file8 (XLSX 13 kb)

## Data Availability

The datasets generated during and/or analyzed during the current study are available in the Array Express repository (accession E-MTAB-3830) and in the SRA (submission: SUB6166902) and BioProject (accession PRJNA559888; SAMN12562584-SAMN12562589) repository. All data generated or analyzed during this study are included in this published article (and its Supplementary Information files).
