# Supplementary material for: miRNAs involved in transcriptome remodeling during pollen development and heat stress response in Solanum lycopersicum
Source: Sci Rep. 2020 Jul 1;10:10694. doi: 10.1038/s41598-020-67833-6 (PMC7329895; doi:10.1038/s41598-020-67833-6)
Supplement: Supplementary file 1 — Supplementary file1 (PDF 778 kb) [file 41598_2020_67833_MOESM1_ESM.pdf]

Supplement to
miRNAs involved in transcriptome remodeling during pollen development and heat stress response in *Solanum lycopersicum*

by

Mario Keller, Enrico Schleiff, Stefan Simm

Supplementary Figures

Supplementary Figure 1: Workflow for the extension of mRNA 3’ ends via MACE
Supplementary Figure 2: Genome browser screenshots of mRNAs with 3’ end extension.
Supplementary Figure 3: Identification and family assignment of miRNAs from small RNA-seq datasets.

Supplementary Tables

Supplementary Table 1: Identified miRNAs in developing and heat-stressed pollen
Supplementary Table 2: Size of detected miRNA families
Supplementary Table 3: Target prediction
Supplementary Table 4: Inversely regulated miRNA-mRNA pairs between the mature and post-meiotic stage
Supplementary Table 5: MapMan terms of upregulated mRNAs between the mature and post-meiotic stage
Supplementary Table 6: Transcription factor families with miRNA-dependent regulation between the mature and post-meiotic stage
Supplementary Table 7: Inversely regulated miRNA-mRNA pairs in response to heat stress

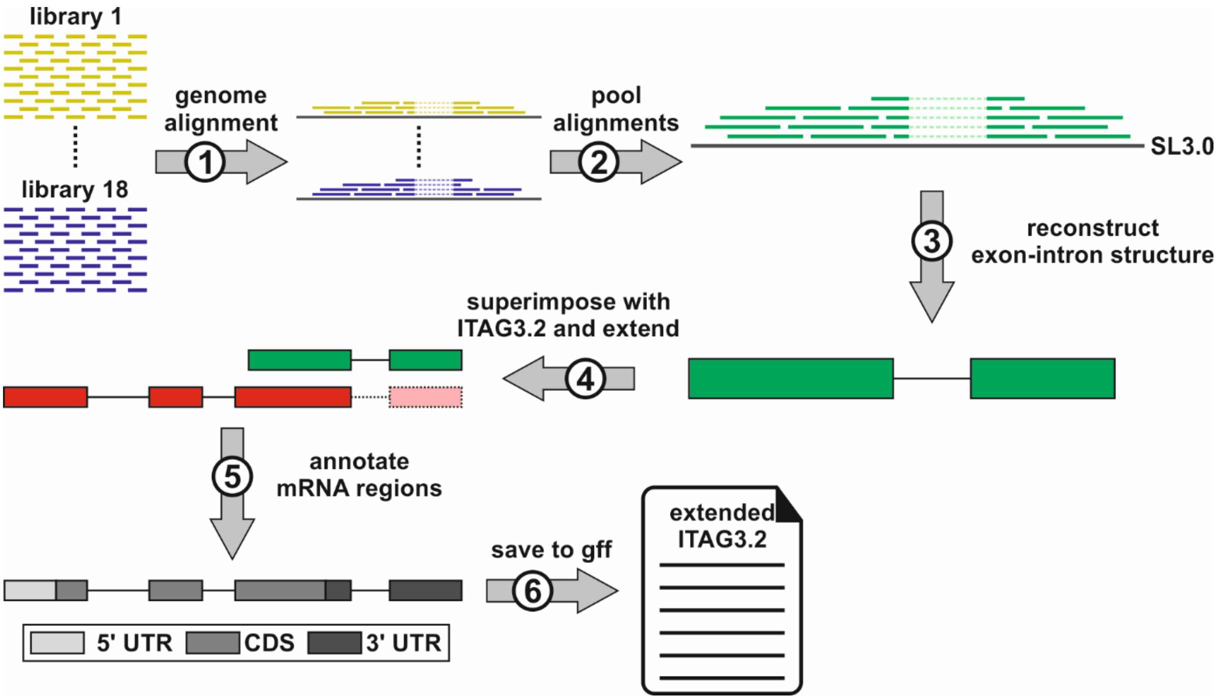

Supplementary Figure 1: Workflow for the extension of mRNA 3' ends via MACE
The workflow consists of six major steps: (1) Sequenced MACE libraries are aligned to the reference genome of tomato (SL3.0), (2) alignments of the 18 libraries are pooled to obtain a higher coverage, (3) continuous and spliced alignments are used to *de novo* reconstruct mRNA 3' ends, (4) reconstructed 3' ends are superimposed on annotated mRNAs of the ITAG3.2, which are extended based on the reconstructed 3'ends if possible, (5) extended regions are annotated as 5'UTR, CDS or 3' UTR, (6) all mRNA structures are stored as extended ITAG3.2 (eITAG3.2) in GFF format.

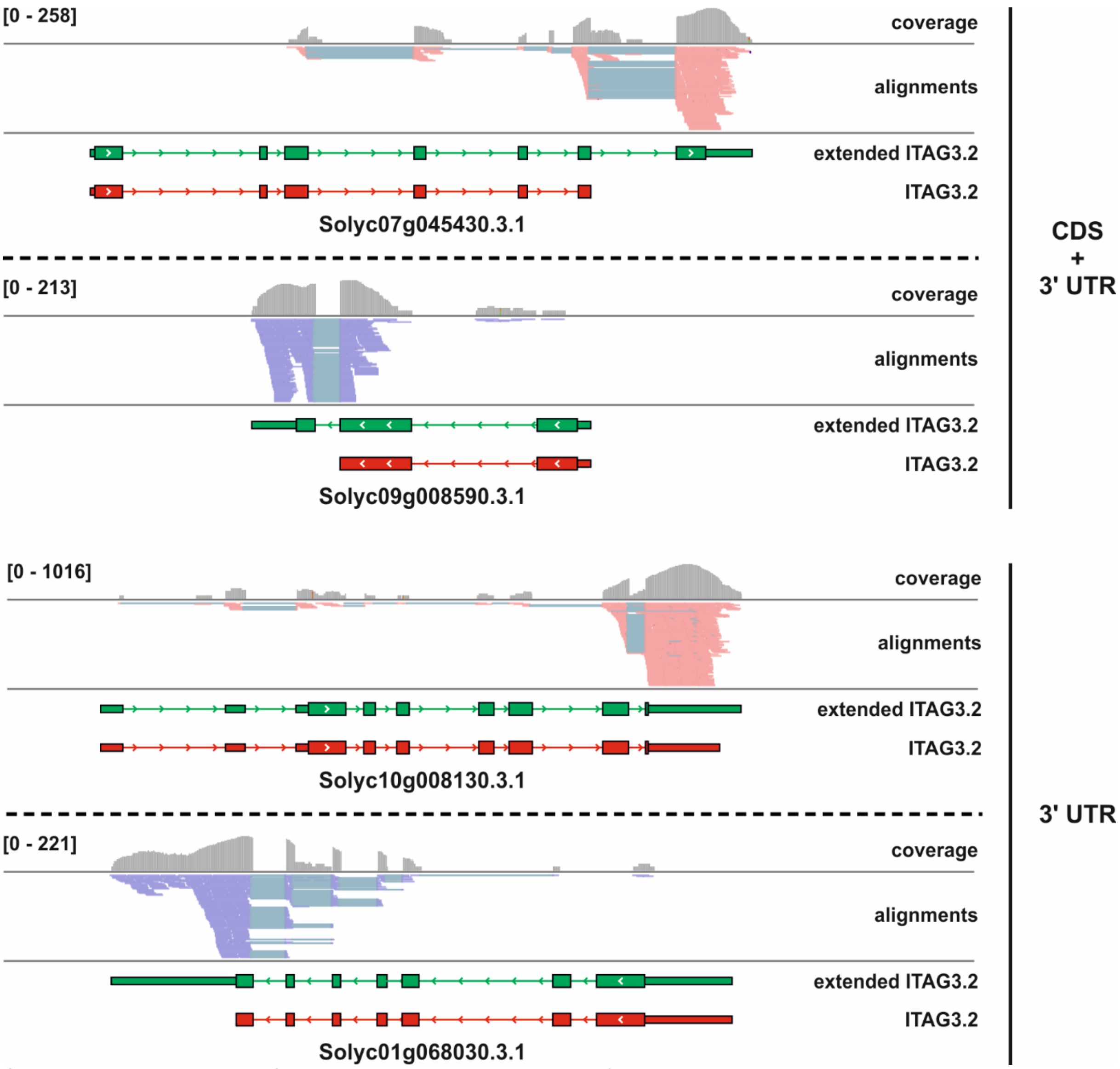

Supplementary Figure 2: Genome browser screenshots of mRNAs with 3' end extension.
Shown are four examples of mRNAs for which an extension of their 3' ends was possible. The two upper panels show examples where an extension of the (incomplete) CDS and 3' UTR was achieved, while in the two lower panels only the 3' UTR was extended. For each example the coverage and alignments of the 18 pooled MACE libraries as well as the original ITAG3.2 and the extended ITAG3.2 annotation are shown.

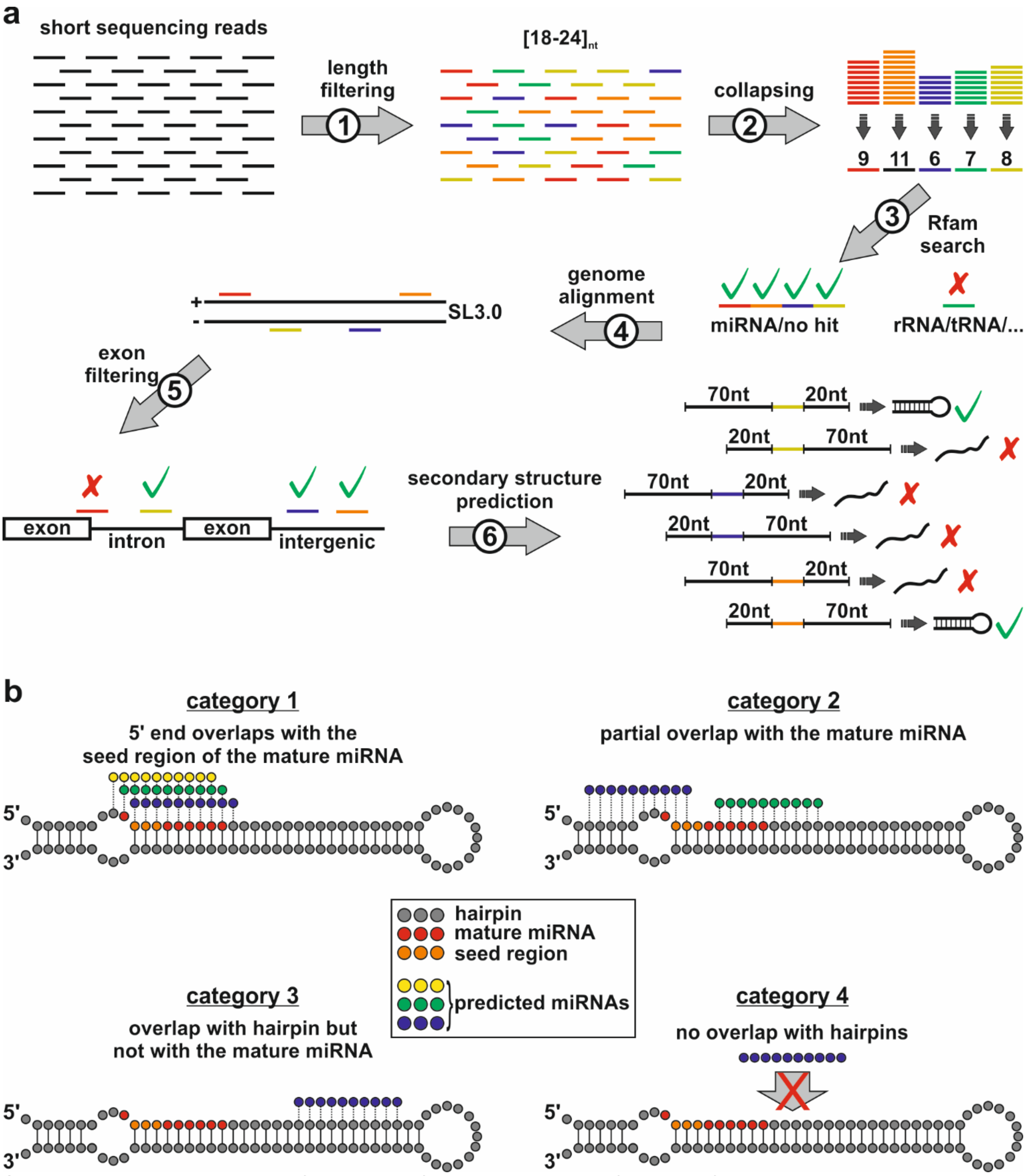

Supplementary Figure 3: Identification and family assignment of miRNAs from small RNA-seq datasets. (a) The workflow for the identification of miRNAs comprises six major steps: (1) Sequencing reads of small RNA-seq libraries are filtered for reads with a length between 18 and 24 nucleotides, (2) identical reads are collapsed to reduce the complexity of the data, (3) sequencing reads are searched against sequences deposited in the Rfam and those matching other RNA types than miRNAs are removed, (4) remaining sequencing reads are aligned to the reference genome of tomato to identify possible genomic locations of the putative miRNAs, (5) alignments overlapping exons of the extended ITAG3.2 are removed, (6) excision of two genomic regions comprising the alignments, which are submitted to secondary structure prediction and subsequent search for hairpin structures. Aligned sequencing reads with at least one excised region folding into a hairpin structure were considered as putative miRNAs. (b) Based on the best-matching hairpin sequences deposited in the miRBase, the putative miRNAs were assigned to miRNA families. The assignment of a miRNA to a family was classified based on four categories: (1) the miRNA 5' end overlaps the seed region of an annotated miRNA on the hairpin, (2) the miRNA has only a partial overlap with an annotated miRNA on the hairpin, (3) the miRNA overlaps with the hairpin but with no annotated miRNA on the hairpin and (4) the miRNA has no overlap with an hairpin (no family assignment).
